# Why Do Consumers Hesitate to Purchase Near-Expiration Food? A Benefit–Risk Perspective on the Green Purchase Paradox

**DOI:** 10.3390/foods15101718

**Published:** 2026-05-13

**Authors:** Xinqiang Chen, Yu Wang, Jiangjie Chen, Chun Yang

**Affiliations:** 1 School of Economics and Management, Xiamen University of Technology, Xiamen 361024, China; chenxq23@xmut.edu.cn; 2 College of Fine Arts, Huaqiao University, Quanzhou 362021, China; chenjiangjie@hqu.edu.cn; 3 School of Design, Jiangnan University, Wuxi 214122, China; 8202201014@jiangnan.edu.cn

**Keywords:** near-expired food, purchase intention, perceived benefits, perceived risks

## Abstract

Near-expired food has received increasing attention in recent years as an important way to reduce food waste and promote sustainable consumption. However, although consumers recognize its economic value and environmental significance, they still have concerns about its quality and potential risks. Drawing on social cognitive theory and social exchange theory, this study adopts a benefit–risk trade-off perspective to examine how personal and environmental factors influence purchase intention toward near-expired food through perceived benefits and perceived risks. Based on 547 valid questionnaires collected from Chinese consumers, this study employs PLS-SEM, multi-group analysis (MGA), and fuzzy-set qualitative comparative analysis (fsQCA) for empirical testing. The results show that personal norm and price discount significantly increase perceived benefits, whereas social image concern and product uncertainty significantly increase perceived risks. Perceived benefits have a significant positive effect on purchase intention, whereas perceived risks have a significant negative effect. The MGA results further show that purchase experience and income level lead to significant differences in consumers’ decision paths. The fsQCA results indicate that both high and non-high purchase intention can be formed through multiple distinct but equivalent paths. High purchase intention mainly follows two patterns, benefit-driven and cognitive trade-off. Non-high purchase intention is mainly characterized by benefit deficiency and risk interference. The findings provide implications for the marketing and risk management of near-expired food.

## 1. Introduction

As concerns over food resource efficiency continue to grow, reducing food waste has become an important issue in global food systems. Reports by the Food and Agriculture Organization of the United Nations indicate that about one-third of food is wasted during production, distribution, and consumption worldwide. This causes major economic losses and also creates serious resource waste and environmental pressure [1,2]. Near-expired food refers to food that has not yet expired but is close to the end of its labelled shelf life. Although the specific near-expiration period may vary depending on product categories and regulatory contexts, such products are generally distinguished from expired food because they remain within the labelled shelf-life period. Near-expired food has gradually been regarded as an important way to reduce food waste. In recent years, with the rise of e-commerce platforms, community group buying, and specialized near-expired food retail channels, the near-expired food market has grown rapidly. Industry data show that the market size of China’s near-expired food industry reached RMB 35.7 billion in 2023 and has continued to expand, with more consumers paying attention to and participating in near-expired food consumption [3]. Compared with conventional food, near-expired food usually has a clear price advantage. It is also seen as a way to reduce food waste and promote resource reuse, giving it both economic and sustainability value.

Although near-expired food has clear price advantages and environmental value, consumers’ evaluations of it remain contradictory. Some consumers regard purchasing near-expired food as a form of sustainable consumption that can reduce living costs and help reduce food waste [4]. Others have concerns about its quality, safety, and freshness and therefore remain cautious about consuming it [5]. In other words, consumers often perceive both potential benefits and potential risks when facing near-expired food. This psychological conflict of recognizing its value while worrying about its risks reflects a typical consumption paradox [6]. In the context of near-expired food, this paradox is particularly evident. Consumers may recognize its price advantage and waste-reduction value while still hesitating because of product uncertainty or social evaluation pressure [7].

Existing research has mainly examined consumers’ attitudes and behaviors toward near-expired food from two perspectives. One line of research focuses on consumers’ understanding of food shelf life and the reasons for refusing to purchase near-expired food. Studies have examined how consumers interpret shelf-life labels and how such interpretations affect their consumption decisions. The findings show that consumers often associate food close to its expiration date with lower quality or potential safety risks, which reduces their acceptance of near-expired food [8,9]. Some studies have further explored the psychological mechanisms behind consumers’ rejection of near-expired food and have shown that concerns about food safety, product quality, and freshness play an important role in attitude formation [10]. Another line of research examines the role of market strategies, especially pricing strategies, in promoting near-expired food consumption. Existing studies generally suggest that price discounts are an important way to increase the attractiveness of near-expired food. Appropriate discount strategies can increase the product’s economic appeal and may also affect consumers’ perceptions of brand quality [11]. Price promotions can also influence consumers’ satisfaction and behavioral intentions, which in turn affect their consumption decisions [12]. More recent research has shown that combining price discounts with messages about reducing food waste can more effectively stimulate consumer demand for near-expired food and promote its acceptance [4]. Existing research has provided useful insights into near-expired food consumption, but some issues still need further investigation.

From a theoretical perspective, existing research has mostly explained near-expired food consumption from single factors or a single theoretical lens and lacks an integrated analytical framework. In practice, consumers’ decisions are often shaped by both personal psychological factors and external environmental factors. Social cognitive theory (SCT) suggests that individual behavior results from the joint effects of personal and environmental factors [13,14]. In the context of near-expired food, personal factors may include personal norm and social image concern, whereas environmental factors may include price discount and product uncertainty. From the perspective of decision-making mechanisms, consumers often weigh benefits against costs when facing consumption choices. Social exchange theory (SET) holds that individuals decide whether to take a certain action based on a comparison of potential benefits and costs [15,16]. In the context of near-expired food, purchase intention is therefore likely to be influenced by the balance between perceived benefits and perceived risks.

From a methodological perspective, existing research has mainly used traditional linear statistical methods to analyze the causal relationships among variables. Consumer behavior, however, is often the result of complex interactions and configurations among multiple factors. Linear relationships have difficulty fully capturing such complex decision-making mechanisms [17]. It is therefore necessary to introduce configurational analysis to gain a more complete understanding of how complex consumer behavior is formed.

Based on the above research background and theoretical gaps, this study takes the question of why consumers remain cautious about purchasing near-expired food even when they recognize its value as the core issue and examines the green purchase paradox in near-expired food consumption. Specifically, this study focuses on the following three research questions:

(1) How do personal and environmental factors influence consumers’ perceived benefits and perceived risks of near-expired food?
(2) How do perceived benefits and perceived risks jointly influence consumers’ purchase intention toward near-expired food?
(3) What combinations of factors can lead to a high level of purchase intention toward near-expired food?

## 2. Literature Review and Research Hypotheses

### 2.1. Theoretical Foundation

SCT, proposed by Bandura [13], is one of the important theories for explaining how individual behavior is formed. It focuses on how internal individual factors and external environmental factors jointly influence behavioral outcomes. It emphasizes that behavior is not determined by a single factor but results from interactions among personal factors, environmental factors, and behavioral outcomes [14]. Within this framework, individuals’ cognition, emotion, and values influence how they interpret external information, while situational cues in the environment in turn shape their cognitive evaluation process and ultimately affect behavioral intention.

Near-expired food consumption also involves the interaction between personal factors and environmental factors. Consumers’ judgments of near-expired food do not come only from product attributes themselves but are also shaped by internal psychological factors and external situational cues [18]. For example, individuals’ sense of responsibility for food waste and their sensitivity to social evaluation influence how they understand the meaning of near-expired food. Price discount, product quality cues, and the uncertainty caused by proximity to the expiration date further shape their cognitive evaluation process [7,19]. Near-expired food consumption is therefore not simply a choice based on product attributes but a judgment process formed by the joint effects of individual psychology and external situations.

However, the perspective of behavior formation alone is still not sufficient to explain consumers’ trade-off process in specific decisions. SET further reveals the decision logic behind consumers’ behavioral choices. This theory was first proposed by Homans [20]. It holds that individuals usually judge whether a behavior is worth taking based on a comparison between benefits and costs during social interaction and behavioral choice. When a behavior can bring higher benefits and lower costs, individuals are more likely to choose it. When a behavior may bring higher costs or risks, their willingness to take that behavior decreases [16].

In the field of consumer behavior, this mechanism of weighing benefits against costs is widely used to explain consumers’ purchase intention and behavioral choice. For example, in product purchase decisions, consumers often consider product price, functional value, and potential risks together and then form an overall evaluation of the product [21]. In the case of near-expired food, this trade-off is particularly clear. Near-expired food usually has a lower price and is seen as helping reduce food waste, which gives consumers both economic value and sustainability value. Consumers may also worry about its quality, freshness, or safety and thus develop a certain level of risk perception [10,22].

### 2.2. Individual Factors and Cognitive Evaluation

Psychological factors at the individual level are considered important variables influencing consumption decisions. Among them, personal norm and social image concern (SIC) are two key factors closely related to sustainable consumption.

Personal norm is usually understood as the moral responsibility or moral obligation that an individual perceives in a specific situation. It reflects an internal requirement regarding how one should act [23,24]. Personal norms may influence consumers’ value judgments. Consumers with a stronger sense of moral responsibility are usually more likely to recognize the social and environmental meaning carried by a certain consumption behavior and are therefore more likely to understand a product from broader value dimensions. For example, Wang, et al. [25] found that personal norm can strengthen consumers’ positive evaluations of sustainable products and environmentally friendly behavior, making them more likely to understand consumption objects from the perspective of environmental benefits and social value. Niu, et al. [26] pointed out in their study of green consumption that personal norm can also, to some extent, buffer consumers’ perceived costs and concerns in green consumption decisions, leading them to make more positive behavioral choices based on environmental benefits even when facing higher personal costs. Shang and Wu [27] further showed in the context of sharing and redistributive consumption that green morality or moral responsibility can enhance consumers’ acceptance of sustainable consumption models and weaken the negative effects of uncertainty and potential costs on behavioral intention.

In the context of near-expired food consumption, stronger personal norms may help increase consumers’ perception of the positive value of near-expired food and may also reduce their sensitivity to related risks to some extent. This study therefore proposes the following hypotheses:

**H1.**  *Personal norm positively influences perceived benefits.*

**H2.**  *Personal norm negatively influences perceived risks.*

Social image concern refers to the extent to which individuals care about others’ evaluations in consumption behavior. It reflects consumers’ sensitivity to how their social image may be affected when making choices [28]. Existing research shows that when consumption behavior may be observed and evaluated by others, consumers pay more attention to whether their choices will affect their image. For example, Islam, et al. [29] show that social influence can significantly shape consumers’ green purchase intention, indicating that consumers’ evaluations of sustainable products are not only based on product attributes but are also influenced by social factors. Liang, Yin and Xu [7] provide more direct evidence in the context of near-expired food. Consumers may avoid near-expired products not only because of concerns about quality or freshness but also because they worry that such purchases may be seen as stingy or a sign of low purchasing power, which creates social evaluation pressure. White, et al. [30] also point out in their review of sustainable consumption that consumers may recognize the environmental or social value of a behavior, but if the behavior does not match the social image they want to present, their behavioral intention may still be reduced.

In the context of near-expired food consumption, stronger social image concern may reduce consumers’ evaluation of the positive value of near-expired food and may also increase their sensitivity to potential social and product risks. Therefore, this study proposes the following two hypotheses:

**H3.**  *Social image concern negatively influences perceived benefits.*

**H4.**  *Social image concern positively influences perceived risks.*

### 2.3. Environmental Factors and Cognitive Evaluation

In addition to individual factors, product attributes and market cues in the consumption environment also influence consumers’ cognitive evaluation. In the context of near-expired food consumption, price discount and product uncertainty are two important environmental factors.

Price discount is one of the most common market strategies for near-expired food. Because near-expired food is close to the expiration date, retailers usually sell it at a discount to reduce inventory loss and improve sales efficiency. Chang and Su [19] found that the level of price discount significantly affects consumers’ perceived value of expiring products, and this effect varies with the perishability of the food. Aschemann-Witzel [31] pointed out in research on suboptimal food that price discount is often one of the most direct drivers of consumers’ acceptance of products close to the expiration date because it strengthens the judgment that the product is worth buying. Theotokis, et al. [11] further showed in their study of expiration date-based pricing that price adjustment changes not only consumers’ evaluation of transaction value but also their overall judgment of product quality. From the perspective of general consumption decisions, consumers’ perception of price is itself an important part of value judgment, and lower prices often mean higher economic value for money [32]. When consumers perceive that near-expired food has a clear price advantage, their perceived benefits are likely to increase.

Price discount may also reduce consumers’ concerns about product risk to some extent. Tsiros and Heilman [22] pointed out in their research on perishable food that remaining shelf life significantly affects consumers’ perceived risk, and price enters consumers’ overall judgment of risks and benefits. Liang, Yin and Xu [7] argued that although price discounts may sometimes reinforce social stereotyping, the discount itself is still a key basis on which consumers evaluate whether near-expired food is worth the uncertainty. A lower price can be seen as compensation for potential risk, which may reduce consumers’ sensitivity to risk. When consumers perceive the purchase cost to be low, their concern about potential loss may also decrease. Szymkowiak, et al. [33] further showed that when consumers understand suboptimal food from a more positive perspective, their negative judgments weaken. This also suggests that price advantage may reduce consumers’ sensitivity to product risk to some extent.

The following hypotheses are proposed based on the above discussion:

**H5.**  *Price discount positively influences perceived benefits.*

**H6.**  *Price discount negatively influences perceived risks.*

Product uncertainty is also an important factor influencing near-expired food consumption. Compared with conventional food, near-expired food is more likely to raise concerns about quality, freshness, and safety. Kavanaugh and Quinlan [34] showed in their study of food date labels that when consumers cannot accurately understand date information, they are more likely to doubt whether the food is still safe. Barone and Aschemann-Witzel [35] further found that when dealing with food close to the expiration date, consumers often rely on date labels or their own sensory judgment, and a lack of trust in labels significantly increases uncertainty. Pandey, et al. [36] also showed in their study of near-expired meat and seafood that consumers’ purchase judgments are strongly influenced by their perceptions of product quality and food safety. When the product condition is difficult to assess, perceived risks are more likely to increase.

Product uncertainty may also reduce consumers’ perceived benefits. Joya and Orth [37] showed that consumers do not share a consistent understanding of food safety, and different types of safety perceptions affect purchase judgments. When consumers cannot form a positive evaluation of product quality, their overall value assessment is affected. Cheng, et al. [38] further showed that when date labels cause confusion, consumers find it more difficult to form a clear judgment about the product condition, which reduces positive evaluation. Cliceri, et al. [39] found that consumers differ in how they understand, attend to, and use date labels, and these differences influence their judgments of product condition and subsequent behavior.

Therefore, this study proposes the following two hypotheses:

**H7.**  *Product uncertainty negatively influences perceived benefits.*

**H8.**  *Product uncertainty positively influences perceived risks.*

### 2.4. Cognitive Evaluation and Purchase Intention

In consumer decision-making, cognitive evaluation is usually regarded as an important mechanism linking external factors to behavioral intention. SET holds that individuals compare potential benefits and potential costs when making behavioral decisions. When consumers perceive that the benefits of a behavior are greater than its costs, they are more likely to adopt that behavior.

In the context of near-expired food consumption, perceived benefits are mainly reflected in two aspects: economic value and sustainability value. Near-expired food usually has a lower purchase cost, and it is also associated with positive meanings such as reducing food waste and improving resource use efficiency. Yang and Chen [40] pointed out that reference groups’ positive perceptions of the economic value and practicality of products can significantly increase purchase or acceptance intention toward suboptimal food. Zhong [41] further found that cognition of the value and meaning of near-expired food is a key mediating link in the formation of reference groups’ purchase intention. A study on the consumption of near-expired dairy products in China also showed that consumers’ positive cognition that near-expired food reduces waste and has practical utility significantly increases their purchase intention [42]. Economic benefits brought by price advantage and sustainability value reflected in waste reduction both strengthen consumers’ positive evaluation of near-expired food and further increase purchase intention. This study therefore proposes the following hypothesis:

**H9.**  *Perceived benefits positively influence purchase intention.*

Perceived risks usually refer to consumers’ subjective evaluation of potential uncertainty in the purchase decision process. In the context of food consumption, perceived risks are often related to food safety, quality stability, and health outcomes. Research on food consumption shows that when consumers perceive higher risks, their repurchase intention and overall acceptance are reduced [21]. Studies on street food also show that perceived risks weaken consumers’ evaluation of product utility and further reduce their subsequent consumption intention [43]. In the context of near-expired food, this effect is more direct. Research on products close to the expiration date shows that consumers often associate proximity to the expiration date with lower quality, reduced freshness, and potential safety problems, which significantly reduces purchase intention [44]. Research on packaging design for near-expired food also shows that consumers’ purchase attitudes toward such products are influenced by their judgments of quality and safety. When risk concerns increase, purchase intention decreases [45]. This study therefore proposes the following hypothesis:

**H10.**  *Perceived risks negatively influence purchase intention.*

### 2.5. Configurational Effects of Purchase Intention

Consumers’ purchase behavior is often not driven by a single factor alone but is more likely to be formed by the joint effects of multiple antecedent conditions. Ragin [46] pointed out that complex social phenomena usually show conjunctural causation and equifinality. Woodside [47] further emphasized in consumer behavior research that purchase behavior does not always follow a single symmetrical causal relationship but is more likely to show multiple paths. Research on online purchase behavior by Pappas, et al. [48] found that high purchase intention is not determined by one single factor but is formed by different combinations of multiple cognitive and emotional factors. Conditions such as perceived benefits, enjoyment, trust, and risk can jointly lead to high purchase intention in different ways. Synodinos, et al. [49] found that consumers’ green food purchase is formed by the joint effects of attitude, subjective norm, perceived behavioral control, and environmental knowledge.

Similarly, the formation of purchase intention toward near-expired food is not determined by a single factor but is more likely to result from the joint effects of personal norm, social image concern, price discount, product uncertainty, perceived benefits, and perceived risks. It is therefore necessary to go beyond the analysis of the net effects of individual variables and further examine the joint effects of different condition combinations on purchase intention toward near-expired food from a configurational perspective.

**Figure 1:**
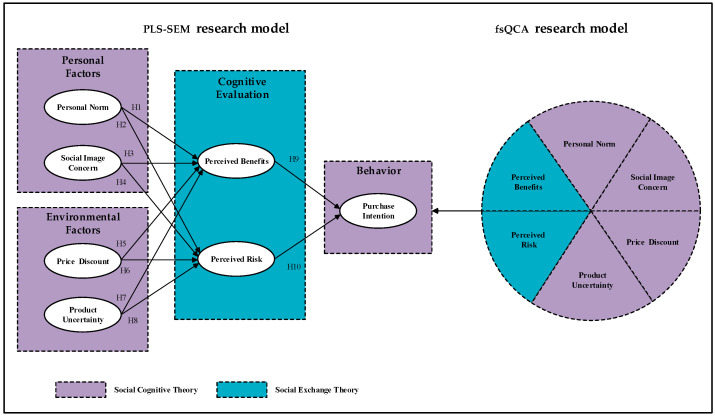
Research model.

The proposed research model and hypotheses are illustrated in Figure 1.

## 3. Research Design

### 3.1. Variable Measurement

This study focuses on the formation mechanism of purchase intention toward near-expired food and includes seven constructs: personal norm, social image concern, price discount, product uncertainty, perceived benefits, perceived risks, and purchase intention. To improve the rigor of the measurement instrument, this study systematically reviewed existing research, selected mature scales from related fields, and adapted some items to fit the context of near-expired food consumption and improve their relevance.

All items were measured using a seven-point Likert scale, ranging from 1 (“strongly disagree”) to 7 (“strongly agree”). A higher score indicates a higher level of agreement with the corresponding statement. The measurement items and literature sources for each construct are shown in Table 1.

### 3.2. Questionnaire Design and Data Collection

The formal questionnaire was developed based on the above measurement items. It consisted of three parts: an introduction to the questionnaire, respondents’ demographic information, and the main measurement items. The demographic section included gender, age, purchase experience, and monthly disposable income. The main section measured all constructs in the study.

This study targeted Chinese consumers and conducted the survey through both online and offline channels, and all data were collected using Tencent Questionnaire. For the online survey, consumers were randomly recruited and received compensation for completing the questionnaire. For the offline survey, participants were mainly recruited through snowball sampling. Before completing the questionnaire, all participants were informed of the research purpose, anonymity, voluntary participation, and their right to withdraw from the study at any time. The whole process, from questionnaire design to data collection, lasted from November 2025 to March 2026. A screening item was included to confirm whether respondents were familiar with near-expired food. Responses from those who were not familiar with near-expired food were excluded from the subsequent analysis. A total of 636 questionnaires were collected. After removing invalid questionnaires with patterned responses or excessively short completion time, 547 valid questionnaires were retained.

Table 2 presents the basic characteristics of the sample. In terms of gender, 308 respondents were male, accounting for 56.3%, and 239 were female, accounting for 43.7%, indicating a relatively balanced gender distribution. In terms of age, the sample was mainly concentrated in the 30–39 age group (302 respondents, 55.2%) and the 20–29 age group (148 respondents, 27.1%). In terms of purchase experience, respondents selecting “Rarely” and “Sometimes” accounted for relatively large shares, at 45.3% and 41.7%, respectively, suggesting that most respondents had some experience with near-expired food. In terms of monthly disposable income, the sample showed a distribution with a higher proportion in the middle-income groups and lower proportions at both ends. The shares of respondents in the 2001–5000 yuan and 5001–8000 yuan income groups were 36.6% and 33.5%, respectively, both higher than those of the other income groups, which generally reflects the income structure of ordinary consumers.

### 3.3. Research Methods

This study employed PLS-SEM, MGA, and fsQCA as the main analytical methods. PLS-SEM was used to evaluate the measurement model and structural model, including reliability, validity, explanatory power, predictive relevance, and path significance, and to test the proposed hypotheses H1–H10. MGA was conducted to examine whether the hypothesized relationships differed across consumer groups and to identify potential group heterogeneity. fsQCA was further applied to explore how different combinations of conditions were associated with high and non-high purchase intention toward near-expired food.

## 4. Results

### 4.1. Common Method Bias Tests

To mitigate potential common method bias (CMB), this study used Harman’s single-factor test to examine the data. The results show that all measurement items yielded five factors with eigenvalues greater than 1. The first unrotated factor explained 27.805% of the variance, which is well below the threshold of 50% [56]. This suggests that common method bias is not a serious concern in this study.

### 4.2. Assessment of Measurement Model

The results of the reliability and validity tests are shown in Table 3. The outer loadings of all measurement items ranged from 0.785 to 0.902, all well above the reference value of 0.7. The Cronbach’s alpha values of all constructs ranged from 0.738 to 0.866, also exceeding the threshold of 0.7 [57]. The average variance extracted (AVE) values of all constructs ranged from 0.655 to 0.788, all clearly above the recommended threshold of 0.5 [58]. The composite reliability (CR) values ranged from 0.743 to 0.867, all above the recommended threshold of 0.7 [59]. These results indicate that the constructs have good internal consistency, reliability, and convergent validity.

In terms of discriminant validity, Table 4 shows that all HTMT values among the constructs are below the threshold of 0.8 [60]. This indicates that the correlations among different latent constructs do not reach the level at which discriminant validity is threatened and that the latent constructs have good distinction. According to the Fornell–Larcker test results in Table 5, the square root of the AVE for each construct is clearly higher than its correlations with other constructs. This indicates that each construct explains its own measurement items better than it overlaps with other constructs [61], which further supports discriminant validity from another perspective. The results from these two methods show that the measurement model has clear boundaries among the constructs and is suitable for structural model analysis.

### 4.3. Assessment of Structural Model

Table 6 presents the assessment results of the structural model, including R^2^, adjusted R^2^, and Q^2^. Q^2^ is used to assess the predictive relevance of the model for endogenous variables. The Q^2^ values of perceived benefits, perceived risks, and purchase intention are 0.362, 0.506, and 0.304, respectively, all greater than 0, indicating that the model has good predictive ability for all endogenous constructs. R^2^ and adjusted R^2^ reflect the explanatory power of the exogenous variables for the endogenous variables. The R^2^ values of perceived benefits, perceived risks, and purchase intention are 0.380, 0.517, and 0.381, respectively. According to the relevant standard [60], the model shows moderate-to-substantial explanatory power for perceived risks and moderate explanatory power for perceived benefits and purchase intention. Overall, the structural model has good explanatory power.

The hypotheses were tested one by one using the bootstrapping method. The results of the path analysis are shown in Table 7. In terms of personal factors, PN has a significant positive effect on PB (β = 0.318, *p* < 0.001), supporting H1. Its effect on PR is not significant (β = −0.034, *p* > 0.05), so H2 is not supported. SIC has no significant effect on PB (β = 0.048, *p* > 0.05), so H3 is not supported. It has a significant positive effect on PR (β = 0.369, *p* < 0.001), supporting H4.

In terms of environmental factors, PD has a significant positive effect on PB (β = 0.406, *p* < 0.001), supporting H5. Its effect on PR is not significant (β = 0.043, *p* > 0.05), so H6 is not supported. PU has a significant negative effect on PB (β = −0.107, *p* < 0.05), supporting H7. It also has a significant positive effect on PR (β = 0.473, *p* < 0.001), supporting H8.

In terms of the relationship between cognitive evaluation and behavior, PB has a significant positive effect on PI (β = 0.605, *p* < 0.001), whereas PR has a significant negative effect on PI (β = −0.258, *p* < 0.001), supporting H9 and H10, respectively.

In addition, the variance inflation factor (VIF) values for all paths range from 1.028 to 1.462, far below the recommended threshold of 5 [62]. This indicates good independence among the explanatory variables and supports the stability of the path estimates.

### 4.4. Multi-Group Analysis (MGA)

After testing the hypotheses in the full sample, most of them were supported. But related research shows that differences in purchase experience and income level may lead to heterogeneity in path relationships among consumers [63,64,65]. It is therefore necessary to conduct multi-group analysis to further examine whether the path relationships differ across groups.

The sample was grouped according to respondents’ purchase experience and income level. Respondents who selected “Never” and “Rarely” were classified as the low-experience group (*n* = 283), whereas those who selected “Sometimes” and “Frequently” were classified as the high-experience group (*n* = 264). In terms of income, respondents in the “2000 yuan and below” and “2001–5000 yuan” categories were classified as the low-income group (*n* = 325), whereas those in the “5001–8000 yuan” and “8000 yuan and above” categories were classified as the high-income group (*n* = 222). Following the recommendation of Henseler, et al. [66], this study used the measurement invariance of composite models (MICOM) procedure to assess measurement invariance across groups in three steps.

The first step examined configural invariance. All groups used the same measurement indicators, questionnaire design, and data processing procedure. The sample sizes also met the requirements for PLS-SEM analysis. The model therefore satisfied the condition for configural invariance. The second step examined compositional invariance. As shown in Table 8 and Table 9, in both the experience-based grouping and the income-based grouping, the original correlations of all constructs were close to 1 and higher than the corresponding 5% quantile values. This indicates compositional invariance across groups and supports the establishment of partial measurement invariance. The third step examined the equality of means and variances. The results show that the original mean differences and variance differences of some constructs fell within the corresponding 95% confidence intervals. The model therefore achieved partial measurement invariance in both group comparisons. Further multi-group analysis could then be conducted.

The results of the multi-group analysis are shown in Table 10. Purchase experience and income level have significant moderating effects on near-expired food consumption decisions. In the experience-based grouping, the effects of PN on PB and SIC on PB are stronger in the low-experience group, indicating that low-experience consumers rely more on moral and social factors in their evaluation. The effects of PD on PB and PB on PI are stronger in the high-experience group, indicating that high-experience consumers pay more attention to economic value. The negative effect of PR on PI is also stronger in the low-experience group, indicating that these consumers are more sensitive to risk.

In the income-based grouping, the effects of PD on PB and PD on PR are stronger in the low-income group, indicating that low-income consumers rely more on price information in their judgment. The effects of PN on PB and SIC on PR are stronger in the high-income group, indicating that high-income consumers are more influenced by norm and social evaluation. The effect of PB on PI is stronger in the low-income group, whereas the negative effect of PR on PI is stronger in the high-income group.

### 4.5. Fuzzy-Set Qualitative Comparative Analysis

The formation of purchase intention toward near-expired food may result from the joint effects of multiple conditions, and complex interactions may exist among different antecedent variables. Traditional net effect analysis is difficult to capture such asymmetrical causal mechanisms. This study therefore further introduced fsQCA to examine the joint effects of multiple condition combinations on the outcome from a configurational perspective. A necessity analysis was first conducted for all antecedent conditions to determine whether any single condition constitutes a necessary prerequisite for high purchase intention or non-high purchase intention. Necessity analysis is mainly assessed by consistency and coverage. Consistency usually needs to reach 0.9 or above for a condition to be considered necessary for the outcome to occur [67].

This study used the direct calibration method [68]. The 5%, 50%, and 95% quantiles of each variable’s data distribution were used as the anchors for full non-membership, the crossover point, and full membership, respectively, to transform the variables into fuzzy-set membership scores ranging from 0 to 1. A single-construct necessity analysis was then conducted on the calibrated data. As shown in Table 11, for both high purchase intention and non-high purchase intention, the consistency values of all conditions and their negations did not reach the threshold of 0.9. This indicates that none of the antecedent variables alone constitutes a necessary condition for high purchase intention or non-high purchase intention.

These results suggest that the formation of purchase intention toward near-expired food does not depend on any single factor alone but is more likely to result from the joint effects and matching of multiple antecedent conditions. Further configurational analysis was therefore needed.

Following the recommendation of Kraus, et al. [69], this study set the analysis parameters as follows: solution consistency no lower than 0.8, PRI consistency no lower than 0.7, and the frequency threshold set at 3. As shown in Table 12, both high purchase intention and non-high purchase intention yielded multiple valid configurational paths. The consistency values of all configurations are above 0.9, indicating good sufficiency of these condition combinations. The raw coverage values of the two types of configurations range from 0.359 to 0.558 and from 0.293 to 0.479, respectively. The overall solution consistency values are 0.933 and 0.938, and the overall solution coverage values are 0.691 and 0.639, respectively, all well above the threshold of 0.2 [70]. This indicates that the configurational results have good explanatory power.

The fsQCA results show that both high purchase intention and non-high purchase intention can be achieved through multiple distinct but equivalent configurational paths. Overall, the configurations for high purchase intention can be classified into two patterns. The first is the benefit-driven pattern, including M1 (PN*PD*PB*~PR), M2 (PN*PD*PU*PB), and M3 (~SIC*PD*~PU*PB*~PR). The common feature of these three paths is that price discount and perceived benefits are both present and mostly appear as core existing conditions, indicating that these two factors are key drivers of high purchase intention. M1 and M3 also show the core absence of perceived risks, indicating that when perceived risks are low, the advantages of price and benefits are more likely to translate into purchase intention. M2 shows that even when a certain level of product uncertainty exists, consumers may still form high purchase intention as long as personal norm, price incentive, and perceived benefits are strong enough. The second is the cognitive evaluation pattern, represented by M4 (~SIC *PD *PU*PB*PR). In this path, price discount and perceived benefits remain important present conditions, while social image concern is absent and perceived risks are present. This indicates that consumers may still form high purchase intention after weighing benefits and risks under a situation in which both are present.

The configurations for non-high purchase intention can be classified into two patterns. The first is the benefit-deficiency pattern, including M5 (~PN*~PD*~PU*~PB*~PR), M6 (~PN*~PD*PU*~PB*~PR), M8 (~PN*SIC*~PD* ~PB*~PR) and M9 (~PN*SIC*~PD*~PU* ~PB). The common feature of these paths is that price discount and perceived benefits mostly appear as core absent conditions, indicating that when consumers cannot perceive the price advantage and benefit value of near-expired food, their purchase intention is more likely to remain low. The second is the risk-interference pattern, represented by M7 (~PN*SIC * ~PD* ~PU*PR * PB). This path shows that even when perceived benefits are present, consumers may still show non-high purchase intention as long as personal norm is insufficient, price discount is absent, and perceived risks are strengthened.

## 5. Discussion

### 5.1. Discussion of the Direct Effects

The results based on SEM show that the formation of purchase intention toward near-expired food is not driven by a single factor alone but results from the joint effects of personal factors, environmental factors, and cognitive evaluation. Perceived benefits have a significant positive effect on purchase intention toward near-expired food (supporting H9), whereas perceived risks have a significant negative effect on purchase intention (supporting H10). The effect of perceived benefits is stronger. This result is generally consistent with existing research on near-expired or suboptimal food consumption. Consumers usually weigh positive benefits such as economic value and waste reduction against potential risks related to quality, safety, and freshness when forming purchase intention toward such food [71,72]. The stronger benefit path in this study suggests that in the context of near-expired food consumption, consumers are more likely to develop purchase intention when they can clearly perceive the price advantage or the value of resource saving. This is also in line with Zhang, van Herpen, Van Loo, Pandelaere and Geuens [4], who found that messages about reducing food waste can increase purchase intention toward near-expired food by enhancing moral satisfaction.

In terms of personal factors, this study finds that personal norm significantly increases perceived benefits but has no significant effect on perceived risks (supporting H1 but not H2). Social image concern does not significantly reduce perceived benefits but significantly increases perceived risks (supporting H4 but not H3). The first result is generally consistent with existing research on food waste reduction and pro-environmental consumption. Personal norm usually strengthens individuals’ sense of behavioral legitimacy and thus promotes related behavioral intention [73,74]. The difference in this study is that, in the context of near-expired food, personal norm mainly works through perceived benefits rather than reducing perceived risks. The unsupported H2 indicates that moral obligation or environmental responsibility does not necessarily reduce consumers’ concerns about quality, safety, or freshness. In other words, consumers may recognize the positive value of near-expired food while still maintaining risk concerns about the product itself. Social image concern does not significantly reduce perceived benefits but significantly increases perceived risks. This suggests that social image concern functions more as a form of negative social evaluation pressure in near-expired food consumption. Consumers may perceive greater risk in purchasing such products because they worry about being labeled as having low purchasing power. This is consistent with Liang, Yin and Xu [7], who argue that social stereotyping strengthens consumers’ resistance to near-expired food. The unsupported H3 further suggests that social image concern does not directly weaken consumers’ recognition of the economic or environmental benefits of near-expired food; instead, it mainly operates by amplifying perceived risks and negative social associations.

In terms of environmental factors, this study finds that price discount significantly increases perceived benefits (supporting H5) but has no significant effect on perceived risks (not supporting H6). Product uncertainty significantly reduces perceived benefits and increases perceived risks (supporting H7 and H8). These results suggest that price discount mainly works as a value cue rather than as a risk-reduction cue. This finding is generally consistent with research on suboptimal food, which shows that discounts can strengthen consumers’ judgments of economic value and value for money and thus increase purchase intention [71,75]. Some studies suggest that discounts may also affect quality judgment or risk associations. This study does not find a significant effect of price discount on perceived risks. This inconsistent result may be explained by the special nature of near-expired food: although discounts improve perceived value, they may not be sufficient to change consumers’ concerns about freshness and safety. In some cases, a low price may even coexist with suspicion about product quality, which could offset any potential risk-reduction effect. This suggests that in the context of near-expired food, consumers interpret price information more as a benefit cue than as a cue that changes risk judgment [11,75].

### 5.2. Discussion of the Multi-Group Analysis

Multi-group analysis further shows that the formation of purchase intention toward near-expired food varies across consumer groups.

In the purchase experience grouping, the path relationships indicate that low-experience consumers are more affected by personal norm, social image concern, and perceived risks, whereas high-experience consumers show stronger links with price discount and perceived benefits. This suggests that consumers with less experience have not yet formed a stable evaluation framework and rely more on normative cognition, social cues, and risk information. Compared with low-experience consumers, high-experience consumers show stronger associations between price discount, perceived benefits, and purchase intention, indicating that economic value plays a more prominent role in their evaluation. This result is generally consistent with existing research showing that acceptance of suboptimal food varies with familiarity, experience, and consumption context [76].

In the income grouping, the path differences suggest that low-income consumers are more sensitive to price discount and perceived benefits, whereas high-income consumers are more influenced by personal norm, social image concern, and perceived risks. This suggests that income level changes the relative importance consumers assign to price, value, and risk information. For low-income consumers, purchase intention toward near-expired food appears to be more price-driven, and clear economic benefits are more likely to translate into purchase intention. For high-income consumers, purchase intention toward near-expired food is more closely related to social evaluation, normative expression, and risk tolerance. This result is generally consistent with research on food choice across income levels, which shows that price and cost factors are usually more important for lower-income groups, whereas higher-income groups are often influenced by more non-price factors [77]. The difference in this study is that, in the specific context of near-expired food, high-income consumers show a stronger negative response to perceived risks. Price advantages may still be offset by risk concerns, which then reduce purchase intention.

### 5.3. Discussion of the fsQCA Results

The fsQCA results reveal the configurational features of the formation of purchase intention toward near-expired food. High purchase intention and non-high purchase intention are not simple symmetrical outcomes but are driven by different combinations of conditions.

For configurations leading to high purchase intention, two main patterns can be identified: a benefit-driven pattern and a cognitive evaluation pattern. The first is characterized by the core presence of price discount and perceived benefits. This indicates that perceived benefits remain a key basis for stimulating purchase intention toward near-expired food. This result is consistent with the net effect results from SEM and with existing research on suboptimal food and near-expired food, which shows that price incentives can significantly improve consumers’ judgments of product value and acceptability, and that the extent to which discounts are translated into clear benefit perceptions is an important condition for purchase intention [71,75,78]. The cognitive evaluation pattern shows that high purchase intention does not necessarily depend on low perceived risks. Consumers may still express high purchase intention when price discount, product uncertainty, perceived benefits, and perceived risks are all present. This suggests that high benefits with risk can also lead to high purchase intention, rather than only the single path of low risk and high benefits.

For configurations associated with non-high purchase intention, the results can be grouped into two patterns: benefit-deficiency and risk-interference. The first shows that when price discount and perceived benefits are both absent, high purchase intention is difficult to form even if some other conditions are present. The second further shows that even when perceived benefits are present, consumers may still maintain non-high purchase intention when personal norm is insufficient, price incentive is weak, and perceived risks are strong. This suggests that the presence of benefits alone is not enough to produce high purchase intention in a stable way. Its effect also depends on whether price stimulation, normative support, and risk conditions are in place at the same time. Existing research often explains barriers to suboptimal food consumption in terms of single factors such as quality concerns, near-expired attributes, or negative associations [5,72]. This study shows that non-high purchase intention is not directly caused by one negative factor alone but is formed by multiple conditions such as insufficient benefits, lack of price discount, and risk interference. Liang, Yin and Xu [7] emphasize the role of social stereotyping in resistance to near-expired food. This study further shows that such negative social cognition is more likely to lead to non-high purchase intention when it is combined with weak benefit support and stronger perceived risks. Non-high purchase intention is therefore not the result of a single linear path but of specific contextual combinations of conditions.

Overall, consumers hesitate to purchase near-expired food not simply because the product is close to the expiration date, but because their decisions are shaped by the joint effects of value attraction, risk concern, and social evaluation. The SEM results show that perceived benefits significantly promote purchase intention, whereas perceived risks significantly reduce purchase intention, with the benefit effect being stronger. This suggests that consumers are willing to consider near-expired food when they clearly perceive price advantages or food-waste reduction value, but this willingness may be weakened by concerns over quality, safety, and freshness. In addition, social image concern significantly increases perceived risks, indicating that hesitation also comes from the worry that purchasing near-expired food may be negatively judged by others. The MGA results further show that this hesitation differs across consumer groups: low-experience consumers are more sensitive to norms, social image, and risk, while high-experience consumers pay more attention to discounts and benefits; low-income consumers are more price- and benefit-oriented, whereas high-income consumers are more affected by social evaluation and perceived risks. The fsQCA results further indicate that high purchase intention can arise when perceived benefits and price discounts are sufficiently salient, but non-high purchase intention may occur when benefit support is weak or when perceived risks interfere with the positive effect of benefits.

## 6. Contributions and Limitations

### 6.1. Theoretical Contributions

This study extends the theoretical perspective on near-expired food consumption. It adopts a benefit–risk perspective and brings value perception and risk perception into a single analytical framework. Previous research has often focused on price or quality as isolated dimensions. This study integrates social cognitive theory and social exchange theory to provide a more complete view of decision-making in sustainable consumption and offers a more integrated perspective for understanding near-expired food consumption.

The study also shows clear heterogeneity in decision-making across consumer groups. The results of multi-group analysis indicate that purchase experience and income level do not merely serve as background variables but shape how consumers respond to price discount, personal norm, social image concern, perceived benefits, and perceived risks. Consumers with different levels of experience and income rely on different evaluation cues, and the corresponding path relationships vary across groups. This highlights the segmented nature of near-expired food consumption and adds to existing research on heterogeneity in consumer behavior.

A configurational perspective is used to examine complex decision-making. By applying fuzzy-set qualitative comparative analysis, the study identifies benefit-driven and cognitive evaluation paths for high purchase intention, as well as benefit-deficiency and risk-interference paths for non-high purchase intention. The results show that high purchase intention and non-high purchase intention are not simple opposites but are produced by different combinations of conditions. This provides a clearer understanding of causal complexity in consumer behavior and offers a method for examining how multiple factors work together in sustainable consumption contexts.

### 6.2. Practical Implications

The findings offer clear practical implications for the near-expired food industry. Firms should combine efforts to strengthen perceived benefits with efforts to reduce risk concerns, rather than relying only on low-price strategies. Price discount remains an important tool for stimulating purchase intention. Firms can make the level of discount more visible, highlight value for money, and emphasize the social value of reducing food waste so that consumers can more easily recognize the benefits of near-expired food. Product uncertainty and social image concern may raise perceived risks. Firms therefore need to improve information transparency by clearly showing the remaining shelf life, storage conditions, and quality assurance information. They can also promote ideas such as rational consumption and green consumption to reduce negative associations with near-expired food.

Firms should also adopt differentiated marketing strategies for different consumer groups. For consumers with less purchase experience or lower income, price advantage and direct benefits should be highlighted more clearly to strengthen purchase motivation. For consumers with more experience or higher income, greater emphasis should be placed on product quality assurance, brand trust, social value, and risk control to increase acceptance. Near-expired food marketing should not rely on one uniform message. It should be designed around the key evaluation cues of different consumer groups.

The configurational results suggest that market promotion for near-expired food is better approached through a combination strategy than through any single factor. In the paths leading to high purchase intention, price discount and perceived benefits repeatedly appear as core conditions. This indicates that shaping perceived benefits should be a basic part of marketing design. In some paths, lower perceived risks also help generate purchase intention. Firms therefore need to reduce consumers’ risk judgments through quality explanations, source traceability, storage guidance, and after-sales support. The results also show that even when some product uncertainty or risk exists, consumers may still form high purchase intention if price incentives and benefit perceptions are strong enough. In practice, firms do not need to remove every concern completely. What matters more is strengthening consumers’ recognition of the product’s overall value.

The paths leading to non-high purchase intention point to two situations that firms should avoid. One is the simultaneous lack of price discount and perceived benefits, which makes it difficult for consumers to recognize the actual value of near-expired food. The other is the case in which consumers perceive some benefits but purchase does not occur because price stimulation is weak, personal norm support is limited, and perceived risks remain high. This means that firms should avoid focusing only on the near-expired attribute without communicating value. They should also avoid relying only on low prices while neglecting risk management. A more effective approach is to combine price presentation, value communication, normative guidance, and risk reduction in a more systematic marketing mix. For platforms and retailers, this means that product pages, in-store displays, promotional messages, and brand communication all need to convey price advantage, practical value, risk protection, and positive consumption meaning at the same time, so that market acceptance of near-expired food can be improved more effectively.

### 6.3. Limitations and Future Research

Although this study offers some theoretical extension and practical implications for research on the decision-making mechanism of near-expired food consumption, it still has several limitations. In terms of data, the study uses cross-sectional survey data, which makes it difficult to fully capture changes in consumers’ cognition and behavior over time. Future research can use longitudinal data for deeper examination. In terms of sampling, this study mainly focused on Chinese consumers. Therefore, the findings may be influenced by China’s specific consumption context, food retail environment, and cultural background. Future research can extend the sample to consumers from other countries or regions to examine whether the decision-making mechanism of near-expired food consumption varies across different cultural and market contexts. In terms of variables, this study focuses on personal norm, social image concern, price discount, and product uncertainty. It does not include other important factors that may affect near-expired food consumption decisions, such as trust, brand perception, and cultural background. Future research can expand the analytical framework on this basis. In terms of method, this study improves explanatory power by combining PLS-SEM and fsQCA. Future research can further strengthen the robustness of the findings by introducing tracking data and big data analysis. In addition, because near-expired food consumption is related to food waste reduction and sustainable consumption, respondents may provide socially desirable answers. Although anonymity and voluntary participation were emphasized during data collection, future research could use experimental methods, behavioral data, or implicit measures to reduce the potential influence of social desirability bias.

## Figures and Tables

**Table 1:** Questionnaire items.

Construct	Items	Source
**Personal Norm** **(PN)**	PN1: I believe that reducing food waste is a responsibility individuals should take. PN2: When edible food is wasted, I feel that it should not happen. PN3: If edible food is thrown away, I feel somewhat uncomfortable.	[50,51]
**Social Image Concern** **(SIC)**	SIC1: I worry that purchasing near-expiry food may influence others’ impressions of me. SIC2: If others see me purchasing near-expiry food, I would feel somewhat concerned. SIC3: I worry that others may interpret purchasing near-expiry food as being “cheap.”	[52,53]
**Price Discount** **(PD)**	PD1: Compared with regular food, near-expiry food usually shows a noticeable price difference. PD2: The pricing of near-expiry food is generally more economical than that of similar products. PD3: The price level of near-expiry food usually feels more cost-effective.	[19,31]
**Product Uncertainty** **(PU)**	PU1: The quality condition of near-expiry food is sometimes difficult to judge. PU2: The actual quality of near-expiry food may involve some degree of uncertainty. PU3: Compared with regular food, near-expiry food is more likely to create a sense of uncertainty.	[44,54]
	MA4: I think the platform sometimes pushes certain videos too aggressively.	
**Perceived Benefits** **(PB)**	PB1: Near-expiry food can help reduce food waste to some extent. PB2: Near-expiry food has certain advantages in terms of economic cost. PB3: Near-expiry food has certain value in terms of environmental protection and resource conservation.	[45,55]
**Perceived Risk** **(PR)**	PR1: Near-expiry food may involve certain food safety risks. PR2: I worry that the quality of near-expiry food may not be as stable as regular food. PR3: Compared with regular food, near-expiry food makes me feel a higher level of risk.	[21,22]
**Purchase Intention** **(PI)**	PI1: If appropriate circumstances arise, I would try purchasing near-expiry food. PI2: I am willing to try purchasing more near-expiry food. PI3: I may continue purchasing near-expiry food in the future.	[44]

**Table 2:** Descriptive analysis of respondents (*n* = 547).

Sample	Category	Number	Percentage (%)
Sex	Male	308	56.3
	Female	239	43.7
Age	20–29	148	27.1
	30–39	302	55.2
	40–49	83	15.2
	50 and above	14	2.6
Experience	Never	35	6.4
	Rarely	248	45.3
	Sometimes	228	41.7
	Frequently	36	6.6
Monthly disposable income	2000 and below	125	22.9
	2001–5000 yuan	200	36.6
	5001–8000 yuan	183	33.5
	8000 yuan and above	39	7.1

**Table 3:** Measurement model analysis results.

Constructs	Items	Loadings	α	CR	AVE
PN	PN1	0.845	0.758	0.758	0.674
	PN2	0.816			
	PN3	0.800			
SIC	SIC1	0.896	0.866	0.867	0.788
	SIC2	0.885			
	SIC3	0.883			
PD	PD1	0.785	0.738	0.743	0.655
	PD2	0.812			
	PD3	0.832			
PU	PU1	0.839	0.796	0.796	0.710
	PU2	0.836			
	PU3	0.853			
PB	PB1	0.827	0.753	0.754	0.669
	PB2	0.802			
	PB3	0.825			
PR	PR1	0.861	0.814	0.814	0.729
	PR2	0.835			
	PR3	0.865			
PI	PI1	0.900	0.866	0.867	0.788
	PI2	0.861			
	PI3	0.902			

**Table 4:** Discriminant validity: Heterotrait–Monotrait ratio (HTMT).

	PN	SIC	PD	PU	PB	PR	PI
**PN**							
**SIC**	0.109						
**PD**	0.724	0.097					
**PU**	0.296	0.522	0.356				
**PB**	0.681	0.074	0.737	0.128			
**PR**	0.150	0.685	0.236	0.791	0.209		
**PI**	0.613	0.109	0.713	0.066	0.695	0.190	

**Table 5:** Discriminant validity: Fornell–Larcker criterion.

	PN	SIC	PD	PU	PB	PR	PI
**PN**	**0.821**						
**SIC**	0.054	**0.888**					
**PD**	0.540	0.078	**0.810**				
**PU**	0.229	0.433	0.275	**0.843**			
**PB**	0.515	0.051	0.552	0.098	**0.818**		
**PR**	0.118	0.575	0.184	0.637	0.164	**0.854**	
**PI**	0.498	−0.095	0.571	0.055	0.562	−0.159	**0.888**

Note: The diagonal of the matrix (boldface) is the square root of AVE.

**Table 6:** R^2^, R^2^ Adjusted, and Q^2^.

Constructs	R^2^	R^2^ Adjusted	Q^2^
PB	0.380	0.375	0.362
PR	0.517	0.514	0.506
PI	0.381	0.379	0.304

**Table 7:** Hypothesis testing results.

Hypothesis	Path	Std Beta	*p*-Value	VIF	Results
H1	PN→PB	0.318	0	1.427	Support
H2	PN→PR	−0.034	0.396	1.427	No Support
H3	SIC→PB	0.048	0.252	1.235	No Support
H4	SIC→PR	0.369	0	1.235	Support
H5	PD→PB	0.406	0	1.462	Support
H6	PD→PR	0.043	0.332	1.462	No Support
H7	PU→PB	−0.107	0.018	1.341	Support
H8	PU→PR	0.473	0	1.341	Support
H9	PB→PI	0.605	0	1.028	Support
H10	PR→PI	−0.258	0	1.028	Support

**Table 8:** MICOM results across experience groups.

Constructs	PZ ^a^	Compositional Invariance		Partial Measurement Invariance	Equal Mean Assessment		Equal Variance Assessment		Full Measurement Invariance
		Original Correlation	5.00%		Original Differences	Confidence Interval	Original Differences	Confidence Interval	
PN	Yes	0.997	0.995	Yes	−0.116	[−0.135; 0.140]	−0.092	[−0.278; 0.275]	Yes/Yes
SIC	Yes	1	0.999	Yes	0.347	[−0.138; 0.138]	−0.554	[−0.213; 0.228]	No/No
PD	Yes	0.999	0.995	Yes	−0.259	[−0.137; 0.146]	−0.027	[−0.223; 0.237]	No/Yes
PU	Yes	1	0.999	Yes	0.154	[−0.141; 0.138]	−0.327	[−0.255; 0.265]	No/No
PB	Yes	1	0.998	Yes	−0.163	[−0.141; 0.143]	−0.181	[−0.225; 0.250]	No/Yes
PR	Yes	1	0.999	Yes	0.181	[−0.145; 0.140]	−0.261	[−0.234; 0.237]	No/No
PI	Yes	1	0.999	Yes	−0.278	[−0.145; 0.137]	0.165	[−0.203; 0.236]	No/Yes

Note: ^a^ indicates configural invariance.

**Table 9:** MICOM results across income groups.

Constructs	PZ ^a^	Compositional Invariance		Partial Measurement Invariance	Equal Mean Assessment		Equal Variance Assessment		Full Measurement Invariance
		Original Correlation	5.00%		Original Differences	Confidence Interval	Original Differences	Confidence Interval	
PN	Yes	0.998	0.995	Yes	−0.177	[−0.146; 0.141]	0.021	[−0.277; 0.298]	No/Yes
SIC	Yes	0.999	0.999	Yes	−0.138	[−0.141; 0.151]	−0.219	[−0.220; 0.219]	Yes/Yes
PD	Yes	0.998	0.995	Yes	−0.148	[−0.150; 0.141]	−0.032	[−0.229; 0.220]	Yes/Yes
PU	Yes	1	0.999	Yes	−0.136	[−0.139; 0.145]	−0.298	[−0.252; 0.258]	Yes/No
PB	Yes	1	0.998	Yes	−0.123	[−0.147; 0.138]	−0.156	[−0.270; 0.246]	Yes/Yes
PR	Yes	1	0.999	Yes	0.043	[−0.155; 0.147]	−0.632	[−0.224; 0.242]	Yes/No
PI	Yes	1	0.999	Yes	0.025	[−0.148; 0.140]	−0.450	[−0.226; 0.230]	Yes/No

Note: ^a^ indicates configural invariance.

**Table 10:** Multi-Group Analysis Results.

Path	Experience Groups			Income Groups		
	Low	High	Difference	Low	High	Difference
PN→PB	0.42 *	0.21	0.21 *	0.219 *	0.442 *	−0.223 *
PN→PR	0.008	−0.078	0.086	−0.02	0.012	−0.032
SIC→PB	0.18 *	−0.052 *	0.232 *	0.053	0.081	−0.028
SIC→PR	0.389 *	0.348 *	0.041	0.243 *	0.514 *	−0.271 *
PD→PB	0.17 *	0.641 *	−0.471 *	0.522 *	0.236 *	0.286 *
PD→PR	−0.08	0.162 *	−0.242	0.173 *	−0.038	0.211 *
PU→PB	−0.174 *	−0.041	−0.133	−0.032	−0.216 *	0.184 *
PU→PR	0.43 *	0.517 *	−0.087	0.449 *	0.406 *	0.043
PB→PI	0.491 *	0.741 *	−0.25 *	0.676 *	0.503 *	0.173 *
PR→PI	−0.373 *	−0.125 *	−0.248 *	−0.156 *	−0.356 *	0.2 *

Note: * indicates *p* < 0.05.

**Table 11:** Necessity Analysis Results.

Constructs	High PI		Not High PI	
	Consistency	Coverage	Consistency	Coverage
PN	0.794	0.779	0.602	0.576
~PN	0.568	0.594	0.769	0.784
SIC	0.690	0.681	0.684	0.658
~SIC	0.654	0.680	0.669	0.677
PD	0.833	0.821	0.573	0.551
~PD	0.545	0.567	0.814	0.826
PU	0.751	0.702	0.689	0.628
~PU	0.603	0.666	0.673	0.725
PB	0.830	0.844	0.539	0.535
~PB	0.543	0.547	0.843	0.828
PR	0.690	0.684	0.688	0.665
~PR	0.661	0.685	0.672	0.679

**Table 12:** Results of Configuration Analysis.

Configuration	High PI				Not High PI				
	M1	M2	M3	M4	M5	M6	M7	M8	M9
PN	▲	●			✖	✖	✖	✖	✖
SIC			△	✖			●	✖	●
PD	●	●	●	●	✖	✖	✖	✖	✖
PU		▲	△	●	△	▲	✖		▲
PB	●	●	●	●	✖	✖	▲	✖	✖
PR	✖		✖	▲	△	△	●	△	
Consistency	0.963	0.930	0.970	0.960	0.943	0.969	0.966	0.950	0.961
Raw coverage	0.461	0.558	0.377	0.359	0.479	0.419	0.293	0.386	0.420
unique coverage	0.011	0.133	0.014	0.020	0.118	0.026	0.018	0.001	0.002
Solution coverage	0.933				0.938				
Solution consistency	0.691				0.639				

**Note. **● indicates core presence; ▲ indicates peripheral presence; ✖ indicates core absence; △ indicates peripheral absence; blank space indicate “don’t care.”

## Data Availability

The data that support the findings of this study are available from the corresponding author upon reasonable request.
